# Combination of Stem Cells with Chinese Herbs for Secondary Depression in Neurodegenerative Diseases Based on Traditional Chinese Medicine Theories

**DOI:** 10.1155/2022/6847917

**Published:** 2022-03-03

**Authors:** Jiahao Feng, Li Liao, Fangfang Xu, Lin Zhang, Jun Zhang

**Affiliations:** ^1^School of Medicine, Sun Yat-sen University, Guangzhou 510632, China; ^2^Hunan University of Chinese Medicine, Changsha 41000, China; ^3^School of Traditional Medicine, Jinan University, Guangzhou 510632, China

## Abstract

Depression is a common secondary symptom in neurodegenerative diseases (NDs) caused by the loss of neurons and glial cells. Recent research focuses on stem cell therapy to replace dead nerve cells, but the low efficiency of stem cell differentiation and short survival time are obstacles limiting the therapy's effectiveness. Clinically, patients with different diseases cannot obtain the same effect by using the same cell therapy. However, traditional Chinese medicine (TCM) often uses syndrome differentiation to determine the treatment plan for NDs. Based on TCM syndrome differentiation and treatment, this article summarizes the advantages of Chinese herbal medicine combined with stem cell therapy, mainly for the effects of various herbs on diseases and stem cells, including prolonging the survival time of stem cells, resisting inflammation, and antidepressant-like effects. In particular, it analyzes the unique pathways of the influence of drugs and acupuncture on different therapies, seeking to clarify the scientific TCM system. This review mainly elaborates on the treatment of secondary depression in TCM and the advantages of a herbal combined stem cell therapy in various methods. We believe it can provide a new clinical concept for secondary depression to obtain good clinical effects and reduce the risks borne by patients.

## 1. Introduction

Depression is a more common secondary symptom related to neurodegenerative diseases (NDs) than sleep disturbances and psychosis [1]. Moreover, neuropsychiatric disturbances are often more problematic and distressing than aspects of NDs to both patients and their families [2]. Zhao et al. [3] reported that depression is present in approximately 40% of people with Alzheimer's disease (AD). Furthermore, many studies have shown that 10–45% of people with Parkinson's disease (PD) have suffered from clinically significant depression [4–6]. Recent studies have explored the relationship between NDs and depression and mechanisms underlying the association between depression and NDs, or co-therapy for both diseases.

NDs are the collective term for AD, PD, Huntington's disease, amyotrophic lateral sclerosis, frontotemporal dementia, and spinocerebellar ataxias [7–9]. NDs are a major threat to human health and are becoming increasingly prevalent due to the growing aging population [10]. The most common NDs, AD and PD, are predominantly observed in elderly individuals, and the risk of these diseases increases with age. NDs have many factors, which include age [11], inflammation [12], reactive oxygen species (ROS) [13], and DNA damage [14]. It is known that aging is a major risk factor for neurodegeneration [9]. Adila Elobeid revealed that the brain tissue from older individuals contains abnormal deposits of aggregated proteins which contain hyperphosphorylated tau (p-tau), amyloid-*β* (A*β*), and *α*-synuclein [15]. However, it remains unclear whether the levels of these deposits are linked with the degree of cognitive impairment and disease progression. When C-X Gong used pseudophosphorylation or the phosphomimicking method to imitate permanent tau phosphorylation, they revealed the structural and functional aspects of the pathologically p-tau in AD's brain and p-tau neurotoxic effects, including caspase activation and initiation of apoptosis [16]. Tau phosphorylation in the proline-rich [17] and C-terminal regions [18] regulates microtubule binding and tau aggregation differentially. Therefore, site-specific tau phosphorylation is one of the culprits of the disease due to p-tau inducing neuronal death. Patients with NDs or secondary depression present with both extracellular A*β* plaques and intracellular tau-containing neurofibrillary tangles in the brain [19]. Many studies have reported that the pathogenesis of NDs relates to changes in A*β* that precipitate the disease process and initiate a deleterious cascade involving p-tau [20]. Therefore, the source of A*β* should be explored. Olige A*β* accumulated in the brains of patients with NDs is thought to be the more toxic species for cells [21, 22] because they can permeabilize cellular membranes, thus resulting in a series of events leading to cell dysfunction and death [23]. Furthermore, amyloid aggregates (low molecular weight) derived from A*β* deposits with redox-active metal ions are considered more toxic since they can produce ROS, deleterious for the normal A*β* peptide and the surrounding biomolecules [24]. The phenomenon will promote the disease progression because the toxic effect constantly causes plaque enlargement and cell autophagy. Increased induction of autophagy involving A*β* is relatively common in NDs, including some disorders in which autolysosome clearance mechanisms are impaired [25]. This increases the neuronal apoptosis rate and accelerates NDs progression. However, many preclinical and clinical studies suggest that a common feature of NDs and secondary depression is dysregulation of the hypothalamic-pituitary-adrenal axis (HPA). Dysregulation of the HPA is caused by A*β* accumulation and tau hyperphosphorylation [26]. Previous studies have shown that A*β* administration can induce dysregulation of the HPA axis [27] and depressive-like behavior in animals [28].

Another common feature of depression and AD is the occurrence of neuroinflammation. Neuroinflammation exists in microglial activation and concentrations of pro-inflammatory cytokines such as IL-1*β*, IL-6, and TNF-*α* in both neurological conditions [29]. The phenomenon can influence the pathogenesis of both depression and AD by interfering with neuronal growth, differentiation, survival, and synaptic activity [30]. This may explain why depression is a secondary symptom in NDs. In a study of AD, microglial cells could remove A*β* plaques [31], but microglial cells also released inflammatory mediators that could contribute to A*β* deposition and further development of plaques due to cell death. In addition, products from dead neurons can further amplify this response by activating toll-like and inducing receptors for advanced glycation end products (RAGE)-dependent activation of p38 mitogen-activated protein kinase (MAPK), thus contributing to A*β*-mediated cortical synaptic dysfunction [32]. In summary, neuronal death is a serious consequence that results from a number of risk factors [33] and is commonly believed to lead to depression [34]. Secondary depression in NDs represents a major threat to human health, so it is necessary to seek treatments to reduce its prevalence.

Nowadays, there is a mainstream view that depression secondary to NDs can be treated by treating the underlying NDs. The treatment of NDs includes drug therapy, gene therapy, and cell therapy. Vinpocetine, a common drug, can decrease the expression levels of serum inflammatory cytokines, TNF-*α*and MCP-1; and increase TLR3 mRNA levels, as well as protein levels of the downstream signaling molecules TRIF-*β*and IRF-3, and serum levels of the anti-inflammatory cytokines IL-10 and IL-8. Meanwhile, it also alleviates cognitive impairment [35] or depression. Furthermore, gene therapy, the delivery of genetic material to supply gene products that will permanently restore abnormal function or bring in target tissues/cells a therapeutic gene [36], has the potential to cure NDs and depression. Nevertheless, drug therapy and gene therapy can only delay the course of the disease or have many limitations. For example, patients are still at risk of NDs because dead nerve cells are rarely replaced by new cells. However, stem cell therapy can avoid this adverse outcome because stem cells will differentiate into nerve cells or other cells. However, differentiations need specific conditions.

The stem cell therapy is widely used and can delay the progression of NDs, plus it appears efficacious for depression. In the treatment of NDs, mesenchymal stem cells (MSCs) are the main stem cell therapy cells used, as they have high differentiation efficiency and safety in patients. Recently, several studies reported that bone-marrow-derived MSCs suppressed T-cell proliferation [37, 38], and the phenomenon may help combat immune rejection during treatment. Inflammation, which plays an important role in NDs and secondary depression in NDs [39], can be relieved by the immunomodulatory effect of MSCs and slows down disease progression. The MSC-induced immunosuppressive mechanism is as follows: (1) MSCs engage the inhibitory molecule PD-1 by its ligands to inhibit T-cell proliferation [40]; (2) MSCs release indoleamine 2,3-dioxygenase (IDO), which inhibits the proliferation of IFN*γ*-producing TH1 cells [41] and blocks NK-cell activity together with prostaglandin e2 (pGe2) [42]; (3) MSCs produce soluble HLA-G5 which can suppress T-cell proliferation, as well as NK-cell and T-cell cytotoxicity, and promote the generation of regulatory T cells [43, 44]. In clinical applications of MSCs, stem cells for tissue repair should easily access the target organ to exert their therapeutic effect through *in situ* administration. However, the method can be hampered by the anatomical location of the damaged tissue or multiple diseased sites [45]. Therefore, the ideal therapy should provide both systemic and local therapeutic effects. Steven M Devine found that MSCs can spread to many tissues after intravenous administration in a study of nonhuman primates using transplantation experiments [46]. Furthermore, other clinical research studies have shown that systemically administered MSCs preferentially migrate to the injury site to support functional recovery [47]. Furthermore, MSCs migrate in response to several chemokines (vascular cell-adhesion molecule 1 and p-selectin) that bind to cognate receptors expressed on their cell surface [48] and lead to the activation of matrix metalloproteinases that degrade the basement membrane [48], accelerate the progression of systemic delivery. In summary, the systemic delivery of MSCs could be a useful way to administer these cells in clinical practice, but we should examine how to enhance stem cell migration and differentiation efficiency.

Traditional Chinese medicine (TCM) refers to the holistic approach to diagnosis, pathophysiology, and therapy in the Chinese herbs, based on accumulated knowledge and practice over a long period of time [49]. TCM has its own theoretical system and application form in stem cell research that emphasizes holism and syndrome differentiation treatment. It has been reported that certain TCM can promote the proliferation and differentiation of stem cells. Compared with other resultants or methods, TCM is relatively safe and easy to be administered in clinical applications. TCM can induce proliferation and differentiation of stem cells and also cause changes in the microenvironment, enhancing stem cell survival and function. Studies have shown that TCM can induce MSCs to differentiate into neural-like cells and modulate immune function to reduce or eliminate immune rejection [50–52]. Using TCM, the concept of holistic medicine, syndrome differentiation, and treatment as guidance, investigation of the mechanisms of proliferation, differentiation, and damage resistance of MSCs can advance the application of MSCs in NDs.

## 2. Mechanism and Advantage of Stem Cells in the Treatment of Secondary Depression

As mentioned earlier, nerve cell death is an important cause of secondary depression in NDs. MSCs primarily originated from bone marrow, and subcutaneous fat can differentiate into neural cells under any conditions [53]. MSCs exist in various tissue systems such as bone marrow, umbilical cord blood, umbilical cord, placenta, amniotic fluid, and adipose tissues [54–56]. MSCs were originally found in the bone marrow, which is also the primary source [57]. They can differentiate into ectomesenchymal cells, including ossification, cartilage, and fat cells [58–60], or non-mesoderm lineages, such as Schwann-like cells that play a role in development, myelination, and regeneration in the peripheral nervous system [61–63]. Therefore, using an injection of MSCs is an effective method that clinicians can use to treat nervous system diseases. MSCs promote other cells to differentiate into neural-like cells but also differentiate into neural-like cells under certain conditions. MSCs promote other cells to differentiate into neural-like cells, but MSCs also induce a proliferation of neural cells [64]. MSCs can produce cytokines, chemokines, and growth factors, which have important roles in creating favorable microenvironments for the proliferation of neural cells at the injury site, enhancing angiogenesis, synaptogenesis, and neurogenesis in the damaged brain tissue [65, 66]. The microenvironment provided by bone marrow MSCs (BMSCs) is beneficial for neural stem cells (NSCs) to differentiate selectively into neuronal and astrocytic phenotype cells. BMSCs can induce neuronal differentiation of NSCs and also enhance neuron survival. Soluble factors secreted by BMSCs are responsible for the neuronal differentiation of NSCs [67]. BMSCs can cross the blood-brain barrier and migrate into the injured site [68] and then differentiate into mesenchymal lineage cells, including neurons and non-neuronal cells in the brain [69]. Jahani et al. [70] indicate that MSCs cultured on nanofiber scaffold can differentiate into neuronal cells. The electrospun scaffolds, particularly scaffolds with random nanofibers, can promote the differentiation of MSCs. Magnetoelectric (ME) nanofibers also comprise the scaffold for the growth of MSCs and differentiation into neural cells because the material can improve the high yield differentiation of MSCs to neural-like cells [71], which is extremely important in treating chronic disease. Furthermore, recent research found that static pressure enables rat MSCs into neural-like cells without bio-factors or chemical disbalance [72]. Studies indicate that cells derived from bone marrow survive, proliferate, migrate, and differentiate into glial and neuronal phenotypes, such as nestin, neurofilaments, MAP-2, or Neu-N [73–78]. Compared with NSCs, MSCs are easy to isolate from the small aspirates of bone marrow that can be obtained under local anesthesia, capable of rapid proliferation in culture, amenable to survival and integration in the host brain, and immunologically inert [79]. MSCs have many advantages in brain injury: (1) inhibition of apoptosis and fibrosis; (2) stimulation of angiogenesis and recovery of blood supply in the lesion [80]; and (3) attenuation of oxidative stress [81]. The MSC-mediated immune regulation mechanism includes suppression of CD4 + and CD8 + T-cell proliferation [82], promotion of regulatory T-cell quantity and enhancement of its immunosuppressive activity [83], and addition to the secretion of immunosuppressive substances [84]. Therefore, MSCs have wide beneficial applications in nerve tissue repair. In clinical practice, doctors always select adipose tissue-derived MSCs (ASCs) to replace BMSCs. Compared with BMSC, ASCs can be more easily isolated, are safer, and considerably larger amounts of ASCs can be obtained [85]. Although injection therapy with ASCs and BMSCs has become the main treatment method for serious tissue damage diseases, the treatment effect can be poor or fail due to unstable ASCs and BMSCs differentiation states. Therefore, we should aim to identify more effective ways to improve the efficiency of cell therapy from other perspectives, for example, TCM.

## 3. Analyzing Stem Cell Therapy of Secondary Depression in NDs Based on the Theory of TCM

“Lingshu·Sea Theory” records that “people have a sea of marrow, a sea of blood, a sea of air, and a sea of valleys…” and the brain is the sea of marrow. This coincides with the main mechanism of differentiation of BMSCs into neuron-like cells in modern medicine. The Lingshu says “At the beginning of generation, the essence is generated first. After the generation of the essence, the brain is formed.” “YixueZhongzongCanxiLu” summarizes its theory as “The brain is the sea of bone marrow, which is generated by the Yang Qi and Yin Qi of the kidney.” The kidney is the congenital foundation, and the main bone produces marrow. The kidney essence is the foundation of the brain and the congenital source of the brain marrow. “Suwen Ni Tiao Lun” records that “if the kidney does not grow, the marrow cannot be full.” The congenital essence hidden in the kidney is nourished by the essence of the water and grain after a person is born to maintain the abundance of the essence of the kidney. The enrichment of the kidney essence also depends on the nourishment of the body's Qi, blood, and body fluid, and the brain marrow is also closely related to the body's Qi, blood, and body fluid. If the Qi and blood are full, the brain marrow will grow. If the Qi and blood are unbalanced and the kidney essence is dysfunctional, the marrow sea will be empty. Insufficiency of the marrow sea can also cause insufficiency of the kidney essence, which affects the body's viscera. Therefore, if the kidney essence fails, the sea of marrow is depleted, and the sea of marrow is filled to strengthen the kidney essence. “ZhuBingYuanHouLun” records that “the kidney stores the essence, and the essence is made by the blood.” Therefore, there is the saying that “essence (Jing) and blood being derived from the same source,” and both essence and blood originate from the essence of the kidney. However, the kidney stores the essence, produces the marrow, and mediates the blood. “Essence and blood are homologous” can also be called “the essence and marrow being derived from the same source.” Therefore, essence, Qi, blood, marrow, and brain form an interactive system. Kidney essence, Qi, and blood are the marrow sea, which is the material basis for brain growth and development. Kidney essence can correspond to embryonic stem cells and the kinds of tissues and organs differentiated from ESCs. MSCs are mainly derived from bone marrow, which has the characteristics of essence, marrow, and blood and can be transformed into other substances. Therefore, people have high expectations for the effect of MSCs in the treatment of neurological diseases. When the Yin and Yang of the Qi and blood of the viscera are unbalanced, it affects the production of innate essence, causing insufficiency of Qi and blood, dystrophy of kidney essence, dysfunction of kidney essence, insufficiency of the marrow sea, and loss of nutrition in the brain, and its physiological functions are naturally affected. This process can also cause nerve cell necrosis or apoptosis and impair the regulation mechanism of stem cell proliferation, migration, and differentiation. Therefore, starting from the theory of TCM, combined with modern medical research, it can be concluded that the regulation mechanism of bone marrow mesenchymal stem cell activation and repair is closely related to essence, marrow, Qi, and blood, and they complement each other and act synergistically. However, secondary depression in NDs, known as “Yu syndrome” in TCM, is mainly caused by emotional injury and stagnation of liver Qi, resulting in a loss of liver relief, loss of spleen health transport, displacement of the heart, and imbalance of Yin, Yang, Qi and blood in the viscera. Based on the principle of “holistic concept, syndrome differentiation, and treatment,” we analyzed the etiology and pathogenesis of secondary depression in NDs. We believe that the main causes are the loss of Qi in the liver and spleen and brain orifices caused by heart displacement and nourishing. Therefore, in the treatment of secondary depression, we should also consider targeted methods based on the TCM theory.

## 4. Application of the TCM Theory Combined with Stem Cell Therapy on Secondary Depression in NDs

Holistic care is important in TCM and the theory emphasizes the unity of both micro- and macroenvironments. Holism has been used in the clinical diagnosis of TCM for a long time, such as syndrome differentiation (Bian Zheng) and treatment. Following the method detailed in Qiao et al. [86], we have divided the Chinese herbal medicines into five categories: tonifying Qi and reinforcing the healthy Qi; tonifying Qi and activating blood circulation; activating blood and resolving stasis; tonifying the kidney to supply essence; and inducing resuscitation by a Fu-unblocking therapy. We have examined those that could be used in the treatment of depression and NDs (Table 1).

### 4.1. Tonifying Qi and Reinforcing the Healthy Qi (TQRHQ)

TCM believes that humans have sufficient positive Qi to resist diseases. On the one hand, positive Qi can stimulate the functions of the Zang-Fu, promote the production of Qi, blood, and other subtle substances of the human body, and improve the metabolism of the microenvironment. Adequate Qi makes it difficult to develop depression or even secondary depression because Qi is related to emotions. TCM doctors have confirmed that *Hedysarum Multijugum Maxim. (huangqi)* and *Panax Ginseng C. A. Mey. (renshen)*, TQRHQ herbs, can promote stem cell regeneration and relieve secondary depression. Astragaloside IV (AS-IV) is a Qi invigorating drug, and *huangqi* has been widely used for the treatment of nervous system diseases in China. *AS-IV* attenuated TLR4 expression through the NF-kB signaling pathway in MSCs, promoting the proliferation of MSCs [87]. In a recent study, *AS-IV* was shown to accelerate differentiation by enhancing the expression levels of nerve growth factor (NGF), which is strongly related to GSK3*β*/*β*-catenin signaling [88]. Further studies showed that the MSCs could differentiate into neurocyte-like and gliocyte-like cells *in vitro* [89]. Both Wnt-1 and Ngn-1 genes play important regulatory roles during the differentiation of rat bone-marrow-derived MSCs to neurocyte-like cells [89]. *huangqi* injection can induce the differentiation of MSCs into neuron-like cells, and the process of differentiation might be mediated by activating Wnt signaling pathways [90]. Wu et al. [91] demonstrated that *ginsenoside-Rg1* from *renshen* could strengthen the spatial learning memory ability in dementia rats with transplantation of BMSCs, which might be possibly correlated to up-regulating mRNA expression of NGF in the basal forebrain after BMSCs transplantation. Yang et al. [92]. found that *astragalus polysaccharide (APS)* effectively reduces mitochondrial ROS accumulation, which can remarkably inhibit apoptosis, senescence, and the reduction of proliferation and pluripotency of BMSCs caused by iron overload [93]. *APS* may play a critical role in the maintenance of the number of MSCs, to ensure the treatment effect when the patient receives the cell therapy. *AS-IV* is also a potential drug against depression as it increases PPAR*γ* expression and GSK3*β* phosphorylation and decreases NF-*κ*B phosphorylation [94]. In *AS-IV*'s functions, the most important mechanism is the upregulation of PPAR*γ* expression because PPAR*γ* expression level affects the inhibition of neuroinflammation. Moreover, as a herb of TQRHQ, *ginseng* also has antidepressant-like effects via ameliorating neuroinflammation and decreasing neuronal apoptosis [92]. Li et al. showed that *ginsenoside Rk1* acts as an antidepressant through its antioxidant activity, the inhibition of neuroinflammation, and the positive regulation of the BDNF-TrkB pathway [95]. Qi can promote the absorption of acquired essence. Acquired essence can replenish the kidney essence, and the kidney essence can be transformed into the marrow, promoting the replenishment of the marrow sea. Patients with secondary depression in NDs possess Qi deficiency, which will hinder the process of marrow replenishment. *Huangqi* and *renshen*, as representative Chinese medicines for TQRHQ, promote the circulation of Qi and the replenishment of marrow (stem cell differentiation).

### 4.2. Tonifying Qi and Activating Blood Circulation (TQABC)

In TCM, Qi can push the blood to move, and Qi deficiency will cause blood stasis. At the same time, the stasis in vessels will further aggravate Qi deficiency, leading to Qi stagnation and secondary depression. Therefore, *TQABC* can promote blood circulation and prevent blood stasis, which is a common method for the treatment of the acute stage of nervous system diseases such as central infarction and cerebral hemorrhage, as well as a TCM method for preventing depression. Recent Chinese medicine studies related to TQABC reported that it induces differentiation of MSCs to neurocyte-like cells. Nie et al. [96] showed the effect of trans-differentiation of MSCs into nerve cells by an ultrafiltration membrane extract mixture from *Angelicae Sinensis Radix (danggui)*, which revealed that BMSCs changed neural-morphologically after induction through upregulation expression levels of neuron-specific enolase (NSE), nestin, NFP, MAP2, glial fibrillary acid protein (GFAP) in the positive control and ultrafiltration membrane extract mixture groups. However, *n-Butylidenephthalide*, an *danggui* extract, was found to be useful in maintaining stem cell pluripotency via the Jak2-Stat3 pathway by inducing cytokine (leukemia induced factor, IL-5, IL-11, et al.) expression levels [97]. *Angelica sinensis polysaccharides*, another component in *danggui*, can significantly up-regulate cyclin D1, RUNX2, OCN, ALP, and BMP-2 protein levels in MSCs. We also found that *Angelica sinensis polysaccharides* activated PI3K/AKT and Wnt/*β*-catenin signaling pathways in MSCs, to promote bone formation [98]. Ha et al. confirmed that *rhodioloside*, which is the main ingredient of *Rhodiola rosea L. (hongjingtian)*, could activate the HIF-1 pathway to promote the survival of BMSCs and repair damaged neurons [99]. Therefore, *rhodioloside* combined with MSCs could be used in the treatment of patients with NDs. We also reviewed herbs that have antidepressant-like effects in TQABC. *Danggui* also exerts antidepressant effects through increasing the level of BDNF protein and increasing the phosphorylation of its downstream targets, which contain cAMP-response element-binding protein (CREB) and extracellular signal-regulated protein kinase (ERK 1/2) in the hippocampus because the phosphorylation of ERK 1/2 and CREB was significantly decreased in the hippocampus of animals with depression [100]. Regarding research of *Rhodiola rosea L.* in depression, these results showed that its extract SHR-5 is efficacious in treating patients with depression [101] and improves depressive and anxiety symptoms [102]. At the same time, its components appear to be well-tolerated with a favorable safety profile compared with conventional antidepressants [103]. However, other herbs of TQABC can improve the symptoms of patients with depression through neurological and rehabilitation effects. Qi can preserve essence, blood can nourish organs and tissues, and blood can transform spirits, so Qi and blood are the main substances in the body's mental activities. Abundant Qi and blood, and unblocked blood vessels, are conducive to the recovery of the sea of marrow and the stability of spiritual sentiment. TCM with the dual functions of invigorating Qi and activating blood can treat secondary depression in NDs caused by Qi stagnation and blood stasis.

### 4.3. Activating Blood and Resolving Stasis (ARBS)

From a TCM viewpoint, the prognosis and rehabilitation of patients with a reduced formation of new blood will be seriously affected due to blood stasis. In the acute stage of brain injury, blood stasis appeared, leading to neuronal necrosis and apoptosis. Blood stasis, similar to thromboses, also severely damaged the regulation mechanism of proliferation, migration, and differentiation of NSCs, increasing the incidence of secondary depression. Herbs of ARBS can usually nourish the blood, remove blood stasis, and promote blood production. Studies have shown that certain Chinese herbs have a specific function of ABRS and have roles in MSCs differentiating into nerve cells, improving central nervous system microcirculation, and assisting in the rehabilitation process after a stroke or brain injury [104]. After induction of *Radix Salviae (danshen)*, MSCs exhibited the typical form of perikaryon with a pyknic cell body and prominence projection neuron. These cells expressed NSE, NF-M, and nestin and were negatively expressed in GFAP [105]. *Salvianolic acid B (Sal B)*, a major bioactive component of the traditional Chinese herb, *danshen*, is widely used in the treatment of cardiovascular diseases [106] and exerts neuroprotective effects [107]. Xu et al. found that *Sal B* had no obvious toxic effects on hMSCs, whereas *Sal B* can promote the osteogenic differentiation of hMSCs by activating the ERK signaling pathway [108]. An extract of *Ginkgo Folium (yinxingye)* named *EGb761* increases stem cells proliferation, but *EGb761* induces stem cell to neural differentiation instead of glial cell differentiation [109]. *EGb761* also provided high levels of neuroprotection, revealing that *yinxingye* for ARBS is beneficial for NDs and secondary depression [110]. Zheng et al. [111] studied the effects of *total saponins of Panax notoginseng (tPNS)* on angiogenesis in rat BMSCs (rBMSCs), and their study showed that *tPNS* (100 *μ*g/ml) can significantly enhance the mRNA expression level of vascular endothelial growth factor (VEGF)-A but no obvious effect on the expression of Flt-1. In Zheng's study, different concentrations of *tPNS* were found to increase the capillary network formation of rBMSCs after Matrigel endothelial differentiation *in vitro* [111]. In studies on *Panax Notoginseng (Burk.) F. H. Chen Ex C. Chow (sanqi)*, the active ingredient of *sanqi* has been reported to have an enhancing effect on osteogenic differentiation of MSCs *in vitro* by upregulating the gene expression level of TGF-*β*1 [112]. *Sodium Ferulate*, as the main active constituent of *Chuanxiong Rhizoma (chuanxiong)*, combined with BMSCs could not only promote expression of glucose transporter 1 (Glut1) and neuron-specific class III beta-tubulin (Tuj1) in the peri-infarct area but also improve BMSCs expression of Glut1, GFAP, and Tuj1, due to up-regulation of stromal cell-derived factor-1 alpha (SDF-1*α*)/chemokine (CXC motif) receptor-4 axis [113]. Similarly, *Polygoni Cuspidati Rhizoma Et Radix (huzhang)* also has the effect of ABRS, which contains a key component *Polydatin* [114]. *Polydatin* can facilitate BMSC migration [115], protect BMSCs from oxidative stress-induced apoptosis [116], promote the neuronal differentiation of BMSCs via Nrf2 activation, and improve neurons functional recovery [117]. This type of herb can also be used in the treatment of depression. *Danshen* positively affects stem cells and the antidepressant-like effect mediated by ERK-CREB-BDNF in the hippocampus [118]. *Yinxingye* and its extracts also have a good effect on depression. Results from both clinical practice [119] and animal models [120] showed that it has an antidepressant-like effect and can effectively improve depressive symptoms by reducing the expression of serum S100B, which is a marker of brain injury. As mentioned earlier, NDs are accompanied by blood stasis, making it difficult for blood to flow smoothly. Blood stasis is blocked in the brain, which further increases the burden of brain tissue; that is, the brain loses nourishment from the blood, and blood stasis will also prevent the essence from becoming marrow to replenish the marrow sea. The lack of marrow in the marrow sea will lead to secondary depression, suggesting that we can avoid secondary depression by promptly using Chinese medicine for ABRS in the early stage of the disease.

### 4.4. Tonifying the Kidney to Supply Essence (TKSE)

According to the liver and kidney homology theory, the liver stores blood, the kidney stores essence, essence, and blood can breed and transform each other, showing that the liver and kidney are fundamental to each other. Liver Qi stagnation can lead to depression because the liver is related to emotions. The kidney stores the kidney essence, which is related to the production of brain marrow, and its essence is equivalent to the function of stem cells. Secondary depression in NDs is caused by nerve cell death and a lack of stem cells. This is similar to the liver abnormalities caused by kidney deficiency and then emotional disorders. The method of TKSE may play an important role in the prevention and treatment of secondary depression. *Rehmannia glutinosa polysaccharide (RGP)* is one of the effective components of *Rehmanniae Radix Praeparata (shudihuang)*, with the effect of TKSE, which can improve the survival rate of stem cells via increasing the p18 expression and decreasing cellular senescence-associated protein p53 and p16 [121]. Furthermore, in mouse behavioral despair depression models, *shudihuang* produced antidepressant-like activities by decreasing serum corticosterone levels, enhancing monoaminergic nervous systems, and upregulating the expression of BDNF or TrkB [122]. It has recently been reported that *Icariin (ICA)*, a major constituent of flavonoids from the Chinese medical herb *Epimrdii Herba (yinyanghuo)*, promotes the proliferation of various types of differentiated cells [123–125]. In the treatment of osteoporosis, *ICA* can promote osteogenic differentiation of MSCs and suppress the formation of adipocyte-like cells. Increased mRNA expression for osteogenic differentiation marker Runx2, osteocalcin, and bone sialoprotein (BSP), and decreased mRNA expression for adipogenic differentiation markers peroxisome proliferator-activated receptor gamma (PPAR*γ*), lipoprotein lipase (LPL), adipocyte fatty acid-binding protein (aP2) occurs. *ICA* inactivated glycogen synthase kinase-3beta (GSK3*β*) and suppressed PPAR*γ* expression is the main mechanism of function [126, 127]. Shuyan Qin's results demonstrate that *ICA* promotes the proliferation of BMSCs through activating ERK and p38 MAPK signaling, which further leads to the up-regulation of their downstream transcription factors Elk1 and c-Myc [128]. *Yinyanghuo* is often suggested as an antidepressant and health product because it is an anxiolytic medicine and is effective in female hormonal disorders [129]. *Lycium barbarum polysaccharide*, from *Lycii Fructus (gouqizi)*, also promotes the generation and development of new neurons and inhibits the MeHg-induced abnormal differentiation of astrocytes [130]. Zhou et al. reported that *Lycium barbarum polysaccharide* might be a potential therapeutic agent for the treatment of NDs against multiple targets that include synaptic plasticity and A*β* pathology due to enhancing neurogenesis [131]. Similarly, *gouqizi* also has the same effect in depression due to its antioxidative properties and decreasing the apoptosis of striatum neuro [132]. *Morroniside*, the main active component of *Cornus Officinalis Sieb. Et Zucc. (shanzhuyu)*, shows abundant biological activities, including anti-apoptosis [133], antioxidative stress [134], and anti-ischemic effects [135]. *Shanzhuyu* can also attenuate hydrargyrum-induced BMSC dysfunction by inhibiting AGE-RAGE signaling and activating Glo1 [136]. Essence is the carrier of sentiment and the foundation of marrowization. The essence of the human body is based on an innate essence, and acquired essence is constantly replenishing and generating. The kidney stores the innate essence and can transform the acquired essence into innate essence. Abundant innate essence can be transformed into marrow to replenish the damaged marrow sea. TCM of TKSE can nourish the kidney and replenish essence, promote the transformation of kidney essence into the marrow, and replenish the marrow of the marrow sea so that the damaged brain tissue can be restored.

### 4.5. Induce Resuscitation by Fu-Unblocking Therapy (IRFT)

In TCM, depression is often related to the internal “heat” of the Fu, such as the stomach, gallbladder, and large intestine. IRFT can remove the “heat” from Fu. The removal of “heat” is equivalent to alleviating inflammation, which can alleviate local inflammation of the brain and slow down the apoptosis of nerves. Therefore, IRFT is useful in the treatment and prevention of secondary depression in NDs. Rhubarb aglycone, a component of *Radix Rhei Et Rhizome (dahuang)*, can decrease the degradation of basal lamina Col IV and the permeability of brain micrangium in cerebral ischemic rats with BMSCs transplantation by regulating the balance of matrix metalloproteinase-9 (MMP-9) and increasing the expression of tissue inhibitor of metalloproteinase-1 (TIMP-1) [137]. *Baicalin*, from *Scutellariae Radix (huangqin)*, has a potent antidepressant activity because of its ability to suppress apoptosis via preventing apoptotic protease-activating factor-1 expression and effectively suppressing caspase-mediated apoptosis signaling cascades [138]. *Honokiol* derived from *Magnolia Officinalis Rehd Et Wils*. *(houpo)*, a famous traditional herb for IRFT, has an anti-inflammation function [139] and fewer adverse effects [140] compared with antibiotics. Importantly, IL-1*β*, a target of *honokiol*, activates inflammatory pathways resulting in a vicious circle of MSCs transplanting and cell survival [141]. In a study of *honokiol* and human umbilical cord-derived mesenchymal stem cells (hUC-MSCs) [142], it was found that *honokiol* relieved these negative impacts induced by IL-1*β* and suppressed the nuclear factor-*κ*B (NF-*κ*B) pathway by downregulating the expression of p-IKK*α*/*β*, p-I*κ*B*α*, and p-p65 in a dose-dependent and time-dependent manner. In Asian countries, *houpo*, used to treat mental disorders, including depression, can increase GFAP mRNA and protein levels to reverse the glial atrophy in the rat brain [143]. Therefore, the combination of anti-inflammation therapy and stem cells could be a novel strategy for better tissue repair. Fu refers to the large intestine, which can relieve patients' accumulated heat and turbidity, makes the blood flow smoothly, and stabilizes the mind. Patients with secondary depression in NDs are often accompanied by dysfunction of Fu and abnormal balance of the middle energizer due to the mutual influence of wind, phlegm, fire, and blood stasis. If the Qi in Fu is not smooth, the heat cannot be evacuated, and the heat will disturb the brain orifice, leading to obstruction of the brain orifice and abnormal emotions. The Fu's Qi is unblocked, and the filthy Qi descends, so the brain orifice opens, and the consciousness is clear. Herbs of IRFT can also purge heat and clear the middle energizer, restoring the circulation of Qi and the blood, removing the heat of the internal organs, and relieving the blockage of the brain.

### 4.6. Chinese Herbal Compound Prescription Agents

Single herbs can be effective in treating diseases, but their side effects can also be more apparent. Therefore, TCM doctors are more inclined to use a range of herbs to treat diseases. Chinese doctors believe that TCM prescriptions can integrate the therapeutic advantages of various herbs and decrease the toxic and side effects. In addition, prescriptions composed of multiple herbs are safer because lower dosages of single herbs contribute to their clinical safety. The TCM prescriptions mentioned below have benefits in preventing and treating secondary depression in NDs*. Shenqi Fuzheng injection (shenqifuzhengzhusheye)*, composed of *Astragalus* and *ginseng*, was proven to induce MSCs to differentiate into neurons *in vivo* in rats with middle cerebral artery occlusion (mcao). Immunohistochemical staining showed that *Shenqi Fuzheng injection* significantly increased the differentiation of MSCs to human NSE, neurofilament (NF), and GFAP [92]. *Buyang Huanwu Tang (buyanghuanwutang)* combined with MSCs transplantation could repair the injured blood vessels and lesion tissues. A study showed that VEGF and Ki-67 expressions were significantly upregulated in the MSCs and combination groups compared to the control and sham operation groups. Moreover, the combination group showed the strongest effect among these groups [144]. In another study of *Buyang Huanwu Tang* [145], it was found that mRNA and protein expression of the neuronal marker, NSE, and neural stem cell marker, nestin, were decreased in BMSCs by treatment with *Buyang Huanwu Tang*, and ERK and p38 in the MAPK signaling pathway were induced to participate in BMSCs differentiation into neuron-like cells. *Naomai Yihao Capsule (naomaiyihaojiaonang)* has the function of TQABC and can resolve phlegm to regulate the “sea of blood in the brain.” The observation of *Naomai Yihao Capsule* combined with BMSCs transplantation showed that *Naomai Yihao Capsule* could promote angiogenesis and neurological impairment recovery by increasing the expression of CD31 in the brain tissue in focal cerebral ischemia rats, which underwent BMSCs transplantation, and the effect was reinforced with extended treatment time [146]. *Xuesaitong capsules (xuesaitongjiaonang)* is one of the main patent drugs used for ABRS, and *Panax notoginseng saponins* is the main ingredient. In rats with cerebral infarction [147], researchers have demonstrated that middle- and high-dosages of *Xuesaitong capsules* can promote and increase the level and mobilization of BMSCs in peripheral blood, which increases the level of stem cell factors and the number of CD117-positive cells and decreases the number of CD54- and CD106-positive cells in the plasma and bone marrow. Despite the long history of TCM prescriptions, few studies have been conducted on the use of stem cells combined with TCM prescriptions. This area is worthy of further investigation.

### 4.7. Acupuncture Combined with MSCs' Therapy

Acupuncture, originating from China, involves the insertion of a fine needle into the skin or deeper tissues at specific locations of the body (acupoints) to prevent and treat diseases [148]. Many clinical studies have shown that acupuncture can effectively promote the functional recovery of neurons after various types of central nervous system injuries (CNSIs) [149]. Its potential mechanisms include the prevention of inflammatory and oxidant stress, suppression of apoptosis, and stimulation of proliferation and differentiation of endogenous NSCs [150, 151]. However, there is an insufficient number of endogenous NSCs capable of differentiating into functional neurons, and the therapeutic effect of acupuncture can be inadequate. In spinal cord injuries, acupuncture promotes neural regeneration and axon sprouting by activating multiple cellular signal transduction pathways, such as the Wnt, Notch, and Rho/Rho kinase (ROCK) pathways [152, 153]. Z. Liu found that the Governor Vessel electro-acupuncture (GV-EA) may activate the process of cell metabolism and initiate synthesis and secretion of endogenous neurotrophic factors in the ambient tissues at the lesion site of the spinal cord [154]. Moreover, based on modified neurological severity scores and immunohistochemistry, a study regarding a cerebral ischemia/reperfusion injury revealed that electroacupuncture and mesenchymal stem cell transplantation interventions are better than MSC transplantation alone as they improve neurological impairment and upregulate VEGF expression around the ischemic focus [155]. However, acupuncture is also used to treat depression. Based on the results of a meta-analysis, acupuncture may be a suitable adjunct to usual care and standard antidepressant medication [156].

## 5. Views and Prospects

For the treatment of NDs and secondary depression, the stem cell therapy has become a major treatment method, but the low survival rate and low differentiation rate of stem cells are still an issue. However, recent studies have shown that Chinese herbal medicine has a positive effect on the survival and differentiation of stem cells and can adjust the inflammatory immune microenvironment and restore dopaminergic nerve function. In particular, the mechanism of action of single or compound components in herbs, inducing MSCs to differentiate into neuron-like cells and its antidepressant effect, is worthy of a more in-depth study. Based on the TCM syndrome differentiation and treatment system, we summarize the mechanism of action of Chinese herbal medicines on disease and the classification of Chinese herbs.

In TCM clinics, patients are defined with a certain type of syndrome, and currently, physicians will prescribe according to the type of syndrome.

**Figure 1:**
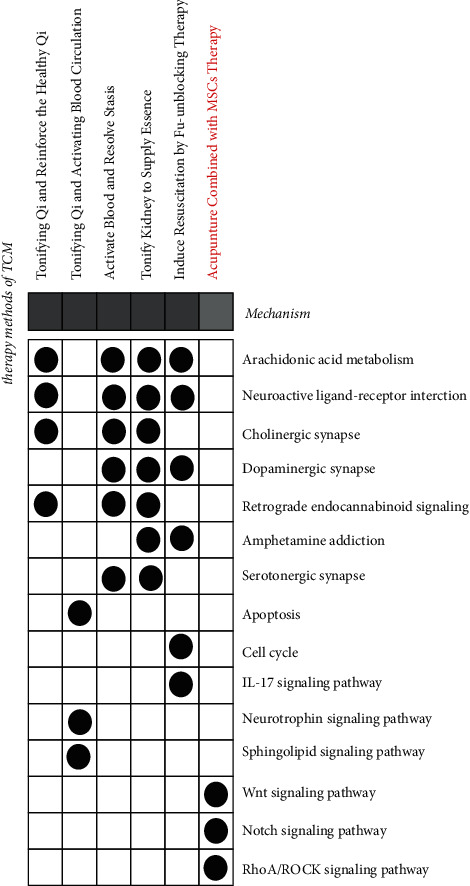
Similarities and differences of pathway enrichment among different treatments. The black dot represents that the treatment method affects the pathway, the right side is the entry of the drug-influenced pathway, and the name of the treatment method is on the top.

The arachidonic acid pathway is involved in neuroinflammatory processes and has protective and detrimental effects in NDs or secondary depression (Figure 1) [157, 158]. This pathway is believed to be related to the excessive activation of microglia, and the excessive activation of microglia is the cause of NDs. Saliba et al. [159] reported that coumarin compounds and their structural analogs can inhibit the cascade effect of the arachidonic acid pathway from slowing down neuroinflammation without involving the endocannabinoid system. These findings show that the combination of TCM components and stem cell therapy can maintain the number of stem cells to ensure efficacy, improve the neuroimmune microenvironment, and reduce the death of nerve cells in regulating the local inflammatory response in patients. The neuroactive ligand-receptor interaction pathway is also a highly relevant pathway for each of our treatment principles. The disorder of this pathway has been shown to be significantly related to NDs and secondary depression [160], and *α*-synuclein can induce miRNA disorders, and miRNA targeting neuroactive ligand-receptor interaction pathways *in vivo* [161]. Among them, miR-133b targets the paired-like homeodomain transcription factor (Pitx3) and regulates neuronal differentiation and activity [162]. MiR-128 can inhibit the transcription factor EB (TFEB) in A9 and A10 neurons, thereby further inhibiting mTOR activation and defense against *α*-synuclein toxicity [163]. The efficacy of drugs with different effects on this pathway can alleviate the toxic effects of *α*-synuclein and affect the differentiation of stem cells into neuron-like cells [164]. This is extremely beneficial for *in situ* stem cell injection therapy of cholinergic synapses, dopaminergic synapses, and retrograde endocannabinoid signaling. Amphetamine addiction is also closely related to the treatment of NDs and secondary depression. These common pathways support the stem cell therapy and focus on eliminating high-risk factors of disease, including inflammation and neuronal cell death, which are beneficial for NDs and secondary depression.

**Figure 2:**
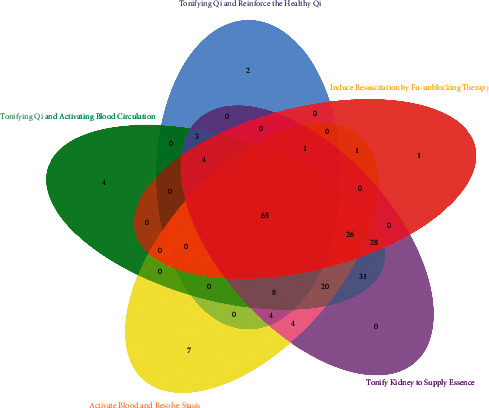
Peculiarities of 5 kinds of treatment methods of TCM enrichment pathways. Different colors represent the drug enriched pathway of different treatment methods, and the intersection part is two or more common pathways.

However, there are special pathways for Chinese medicines of different treatment methods (Figure 2 and Table 2). In TQABC, there is an apoptosis and neurotrophin signaling pathway. We found the clinical phenomena that the use of blood-activating drugs in patients with cerebral infarction can protect the brain nerves of the patients and promote the effects of the patients' volatilization [165]. IRFT is related to the cell cycle pathway and IL-17 signaling pathway. The cell cycle pathway can promote cell proliferation and constant number [166]. IL-17 is one of the working cytokines of the inflammatory storm [167]. Its level is significantly related to apoptosis. This is not available in other syndrome types. Therefore, this type of TCM is extremely beneficial to the survival of the stem cells and the suppression of the secretion of local inflammatory factors after stem cell therapy. Thermogenesis is a unique pathway for TQRHQ treatment, which is related to the formation of NDs because this pathway is related to the activity of mitochondria, and changes in mitochondrial redox affect cell proliferation and apoptosis [168]. The main characteristics of patients with depression and Qi deficiency syndrome are low immunity, malnutrition, or a long course of the disease, which leads to insufficient vitality. This phenomenon is related to insufficient body energy supply and insufficient cell replenishment, which is compatible with the special pathway of TQRHQ. In TQABC, the pathways of fructose and mannose metabolism, glucagon signaling pathway, starch and sucrose metabolism, and pathways related to sugar metabolism have also been shown to have a negative impact on the nerves. The progress of degenerative diseases has also contributed [169]. In particular, the glucagon pathway affects blood glucose levels. The level of blood glucose directly affects the processing of A*β* precursor protein toxicity and the clearance of A*β* [170]. Therefore, this type of TCM can effectively intervene in the progression of NDs caused by metabolic syndrome. Regarding patients with secondary depression of Qi stagnation and blood stasis type, the disorder of nutrient metabolism is similar to the deficiency of Qi in TCM. The accumulation of A*β* is the manifestation of blood stasis. Therefore, the special pathway of TQABC is extremely important for the imbalance of the body because this type of herb can interfere with diseases by affecting blood sugar. In the analysis of ABRS, the pathway of aldosterone synthesis and secretion is only present in the treatment. Single nucleotide polymorphisms and altered gene expression of components of the renin-angiotensin system are associated with secondary depression in NDs [171]. Renin-angiotensin-aldosterone system (RAAS)-activated angiotensin-type (AT4) receptor is also beneficial in NDs [172]. Its intervention in histidine metabolism may also be a promising therapeutic target for brain diseases to restore patients' circadian rhythms and solve sleep-wake disturbances [173]. From the perspective of TCM, the congestion degree of blood stasis syndrome is stronger than that of Qi stagnation and blood stasis and is accompanied by blood deficiency. Hypokalemia caused by high aldosterone levels has similar symptoms to blood deficiency, with clinical phenomena such as fatigue and numbness of the limbs. Aldosterone is related to local tissue remodeling and fibrosis and participates in inflammation, as is that abnormal aldosterone represents blood stasis in patients, so ABRS is more effective for patients with secondary depression with blood stasis syndrome. The most unique of IRFT is the tight junction. Tight junctions affect the stability of the blood-brain barrier, and the abnormality of tight junctions may trigger and/or contribute to a “vicious circle” such as neurodegenerative disease processes [174]. This pathway is also closely related to inflammation [175]. It is possible to build a therapeutic system together with the stem cell therapy by maintaining the stability of the blood-brain barrier and reducing inflammation. As a targeted method, these pathways are particular to secondary depression with different syndromes in TCM, ensuring a good curative effect.

Combining TCM theories of medicinal properties, pharmacodynamics, and holism with western scientific approaches would help to advance the efficacy of TCM. Combining the concepts of TCM with modern medicine and technology will promote novel treatment ideas.

## Figures and Tables

**Table 1:** Function and classification of traditional Chinese medicine.

Treatment rules	Herb or patent drug	Component	Function
Tonifying Qi and reinforce the healthy Qi	Huangqi	Astragaloside IV	Promotes proliferation and differentiation of stem cells; antidepressant-like effects
		Astragalus polysaccharide	Reduces mitochondrial ROS accumulation and iron overload
	Renshen	Ginsenoside-Rg1	Upregulates mRNA expression of NGF
		Ginsenoside Rk1	Antidepressant-like effect
	Shenqi Fuzheng injection		Promotes differentiation of stem cells

Tonifying Qi and activating blood circulation	Danggui	*n*-butylidenephthalide	Maintains stem cell pluripotency; antidepressant-like effect
		Angelica sinensis polysaccharides	Promotes differentiation of stem cells
	Hongjingtian	Rhodioloside	Increases the survival of stem cells; antidepressant-like effect
	Buyang Huanwu Tang		Promotes differentiation of stem cells
	Naomai Yihao capsule		Promotes angiogenesis and neurological recovery

Activating blood and resolving stasis	Danshen	Salvianolic acid B	Promotes differentiation of stem cells; antidepressant-like effect
	Yinxingye	EGb761	Promotes differentiation of stem cells; antidepressant-like effect
	Sanqi	Total saponins of panax notoginseng (tPNS)	Promotes differentiation of stem cells
	Chuanxiong	Sodium ferulate	Promotes differentiation of stem cells
	Huzhang	Polydatin	Facilitates stem cells migration; reduce oxidative-induced apoptosis
	Xuesaitong capsules	Panax notoginseng saponins	Promotes mobilization of stem cells; regulate the immune environment

Tonifying the kidney to supply essence	Shudihuang	Rehmannia glutinosa polysaccharide	Increases the survival of stem cells; antidepressant-like effect
	Yinyanghuo	Icariin	Promotes proliferation of stem cells; regulate female hormonal imbalance; antidepressant-like effect
	Gouqizi	Lycium barbarum polysaccharide	Promotes the generation of neurons; antidepressant-like effect
	Shanzhuyu	Morroniside	Anti-apoptosis effect; antioxidative stress effect; anti-ischaemic effect; attenuate stem cells dysfunction

Inducing resuscitation by a fu-unblocking therapy	Dahuang	Rhubarb aglycone	Promotes transplantation of stem cells
	Huangqin	Baicalin	Anti-apoptosis effect; antidepressant-like effect
	Houpo	Honokiol	Anti-inflammation function

**Table 2:** Peculiar pathway of governance.

Governance	Special pathway
Tonifying Qi and reinforce the healthy Qi	Thermogenesis

Tonifying Qi and activating blood circulation	Fructose and mannose metabolism
	Glucagon signaling pathway
	Starch and sucrose metabolism

Activating blood and resolving stasis	Aldosterone synthesis and secretion
	Glycine, serine and threonine metabolism
	Histidine metabolism
	Phenylalanine metabolism

Induce resuscitation by fu-unblocking therapy	Tight junction

## Data Availability

No data were used to support this study.
